# Risky Trade-offs: Bangladeshi Quest for Safe Water

**Published:** 2004-08

**Authors:** M. Nathaniel Mead

In an attempt to eliminate epidemic levels of diarrhea and other infectious diseases associated with the use of surface waters, millions of shallow tube wells were drilled into the Ganges Delta alluvium in Bangladesh beginning in the early 1970s. This process reduced the rates of water-related infectious diseases but created a new public health dilemma: a surge in diseases such as skin ailments, diabetes mellitus, and various cancers, all resulting from habitual consumption of groundwater naturally high in arsenic.

A number of interventions have been proposed to help remedy the widespread arsenic exposure, but these interventions may only be bringing the catastrophic water situation in Bangladesh full circle. A new study by epidemiologists led by Kamalini Lokuge of the Australian National University suggests that, while these interventions will eventually result in less disease overall, they may initially cause a steady and considerable increase in diarrheal disease **[*EHP* 112:1172–1177]**. The study indicates that any large-scale transition away from household tube wells as a source of drinking water, without proper evaluation of the risks, may be premature.

In attempting to quantify the disease burden resulting both from arsenic exposure and from the potential side effects of widely available arsenic mitigation interventions, Lokuge and her colleagues used previously published information to estimate mortality rates and disability-adjusted life years (DALYs). Simply put, a DALY is a measure of the burden of disease; it reflects how much a person’s expectancy of healthy life is reduced by premature death as well as by disability caused by disease.

The Australian team used World Health Organization data to estimate the DALYs lost per year to arsenic-related effects including diabetes, ischemic heart disease, and a number of cancers. They calculated that arsenic exposure causes the loss of 174,174 DALYs per year in Bangladeshis exposed to arsenic concentrations above 50 micrograms per liter (μg/L), the nation’s cut-off point for safe drinking water.

Then they calculated the DALYs that would be lost to infectious disease, provided Bangladeshis adopted certain arsenic mitigation options currently advocated by the federal Bangladesh Arsenic Mitigation and Water Supply Project and immediately accessible to the majority of the Bangladeshi population year-round. These include surface water supplies, uncontaminated community tube wells, and low-cost filtration systems. These alternative options carry the potential for increased water-related infections, compared with household tube wells.

Assuming that mitigation efforts were undertaken only in those areas where the arsenic concentration of drinking water is highest (100–300 μg/L), the team found that the long-range benefits of arsenic mitigation in terms of DALYs gained and deaths avoided would outweigh any initial decline in public health due to water-related infectious diseases. However, there would initially be a period of some years (the number of which is still unknown) before any benefit would accrue, and some additional years until the total benefit outweighed the cost of the water-related infectious disease increase. The investigators also conclude, moreover, that if the Bangladeshi people gradually stop using the alternative water sources and processes (for example, because of the inconvenience of maintenance or complacency as disease drops off), the initial DALY-based cost of water-related infectious diseases would remain while the long-range benefits would disappear.

The study demonstrates that implementation of any arsenic-mitigating intervention must take into account not only the strategy’s effectiveness in reducing arsenic exposure but also its safety in terms of water-related infectious diseases, the likelihood of population-wide compliance, and different exposure levels within the population. The investigators contend that such information is vital to developing appropriate policies toward resolving the drinking water crisis in Bangladesh.

